# Supplementary material for: Comparative multiomics analysis of cell physiological state after culture in a basket bioreactor
Source: Sci Rep. 2022 Nov 23;12:20161. doi: 10.1038/s41598-022-24687-4 (PMC9686226; doi:10.1038/s41598-022-24687-4)

3666056.1:39036366:39036513:+@NW_023666056.1:39036642:39036787:+@NW_023666056.1:39038357:3903[illegible]

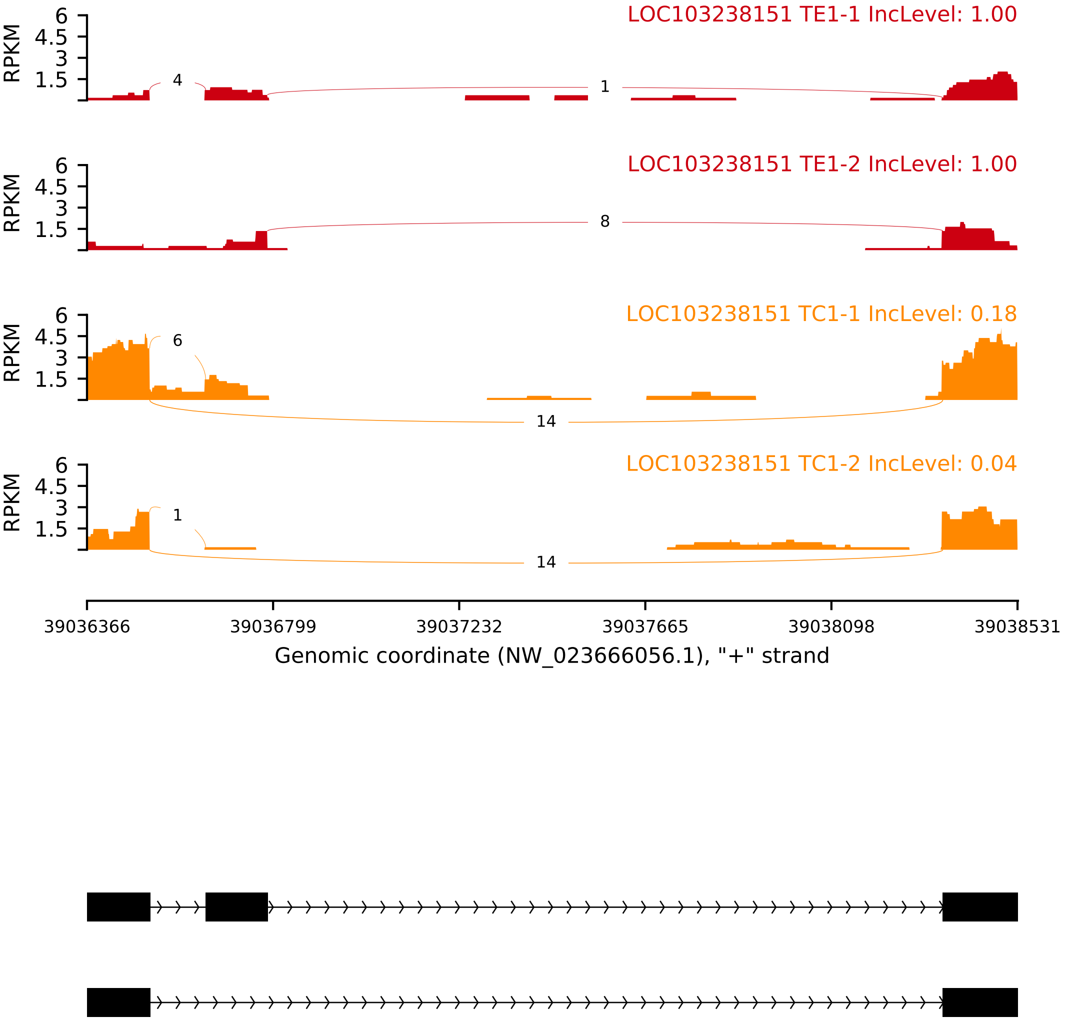

Supplement: Supplementary file 1 — Supplementary Information 1. [file 41598_2022_24687_MOESM1_ESM.zip › raw data/Transcriptomic raw data/7.AS/2.dasplot/TE1vsTC1/SE/17_LOC103238151_NW_023666056.1_39036366_39036513_+@NW_023666056.1_39036642_39036787_+@NW_023666056.1_39038357_39038532_+.pdf]
